# Optimization of Activated Carbon Synthesis from Spent Coffee Grounds for Enhanced Adsorption Performance

**DOI:** 10.3390/molecules30122557

**Published:** 2025-06-12

**Authors:** Geon-Woong Hyeon, Gi Bbum Lee, Da Jung Kang, Sang Eun Lee, Kwang Mo Seong, Jung-Eun Park

**Affiliations:** 1 Bio Resource Center, Institute of Advanced Engineering, 175-28 Goan-ro 51 beon-gil, Baegam-myeon, Cheoin-gu, Yungin-si 17180, Gyeonggi-do, Republic of Korea; 7669woong@iae.re.kr (G.-W.H.); mnbbv21c@iae.re.kr (G.B.L.); kangdj1119@iae.re.kr (D.J.K.); lse9907@iae.re.kr (S.E.L.); 2 Sustainable Materials Research Team, Hyundai Motor Group, 37, Cheoldobangmulgwan-ro, Uiwang-si 16082, Gyeonggi-do, Republic of Korea; aektu2000@hyundai.com

**Keywords:** spend coffee grounds, activated carbon, high specific surface area, mesopore volume, butane working capacity

## Abstract

As an adsorbent, biomass activated carbon is effective at the removal of a wide range of organic and inorganic pollutants; however, its synthesis remains complex. Although spent coffee grounds (SCG) could be an effective material for the production of activated carbon, achieving a sufficient surface area has proven to be difficult. Here, this study presents a preliminary investigation into the optimal manufacturing conditions of activated-carbon adsorbents derived from SCG. SCG samples were characterized according to proximate analysis, elementary analysis, surface area, and pore volumes, then subjected to various processes (i.e., drying, carbonization, and chemical activation) with different operating parameters (temperature and time). The samples were optimized as follows: (1) Stable drying of SCG with a high moisture content of approximately 65% required consumption energy of 49 kWh/kg and drying at 105 °C for 20 h. (2) By comparing changes in the consumption energy and product yield with an increasing amount of carbon fraction, it was found that drying carbonization was more suitable than hydrothermal carbonization for SCG. The optimum drying carbonization temperature for achieving attractive biochar was 500 °C for 1 h. (3) Activated carbon with the optimum surface area (3687 m^2^/g) and mesopore volume fraction (approximately 70%) was achieved with a chemical activator agent ratio of approximately 3 and heating at 850 °C for 1 h. Furthermore, the butane working capacity of the activated carbon was related to the mesopore volume/surface area and reached 74.5% at a mesopore volume/surface area of 0.0004, indicating its suitability for activated carbon canisters. These findings can be used to optimize the synthesis of industrial-grade activated carbon from SCG.

## 1. Introduction

Coffee is a popular beverage worldwide, with its production reaching 10 million tons in 2019. In 2023/2024, global coffee consumption grew by 2.2% from the previous year to 176 million bags, following an average annual growth rate of 0.6% since 2017 [1,2]. The global production of instant coffee and coffee brewing generates approximately six million tons of spent coffee grounds (SCG), with this outcome being increasingly highlighted as an environmental issue [3,4].

Coffee bean brewing produces an estimated 98% residual coffee waste and 2% coffee. Residual coffee waste includes 60% liquids (moisture, oils, etc.) and 40% solids (spent coffee grounds, SCG) [5,6]. According to the World Bank, in high-income countries, approximately 39% of waste is landfilled, 36% is recycled or composted, and 22% is incinerated for energy recovery [7]. However, SCG dumping in high-load landfills drives air pollution via the emission of CO_2_ and CH_4_. Additionally, SCG dumping has also been shown to drive water pollution through the release of phenol, chlorogenic acid, hydroxycinnamic acid, and quinin acid. Therefore, demand is increasing for new methods of treating or recycling SCG as an alternative to landfilling [5,8].

Biomass has been shown to be useful in the production of liquid fuel [9], solid fuel [9,10], building materials [9,11,12], cosmetics [13], and adsorbents [14,15,16,17,18,19,20,21]. There has been a concerted global effort to shift toward biofuel and biochemical alternatives to fossil fuel. From the perspective of SCG, numerous studies have attempted to overcome the low yield of SCG in the bio-ethanol process and its high energy consumption in the bio-diesel process, as SCG contains high contents of carbohydrates and lipids when converted to bio-ethanol (0.15 g/g-SCG) or bio-diesel (0.22 g/g-SCG) [9]. Moreover, biomass solid fuel such as briquettes and pellets can be manufactured from compressed SCG, which contains a similar energy density to wood chip (20.1 MJ/kg) [10]; however, the direct combustion of SCG may lead to air pollution from nitrogen oxide and sulfur oxide. As a packing and building material, SCG feedstock exhibits good physical properties (e.g., tensile strength and impact resistance) that can be enhanced by approximately 10–20% by varying the particle size and distribution [11]; however, consistent application is hindered by technical limitations [9,11,12]. Furthermore, owing to its rough texture, SCG feedstock can also be used to produce cosmetics, such as scrubs and exfoliators, and has potential as an alternative to microplastics [13]. However, technical problems arise in relation to reducing residual caffeine and ensuring skin safety.

Many existing adsorbents either are made of relatively poor-quality materials or involve complex production, culminating in their commercialization being hindered by the high energy consumption and low product yields [14,15]. Conversely, biomass activated carbon (BAC) is a highly effective adsorbent that boasts a high surface area over 2000 m^2^/g as well as heterogeneity, making it effective at the removal of various organic and inorganic pollutants. However, various thermal and biochemical processes and combinations of processes have been proposed for manufacturing BAC, which affects the resulting adsorbent quality [15].

The many activation methods for producing BAC from SCG include three main stages: drying, carbonization, and activation. First, complete drying is required before carbonization because of the high moisture content of SCG. Second, SCG carbonization is performed at 300–700 °C to increase carbon content and reduce organic materials via thermal treatment in an oxygen-free environment [4,16]. However, recent studies have demonstrated that applying higher pyrolysis temperatures, such as 850 °C, can further improve the porosity and structural properties of the resulting biochar, albeit with increased energy consumption [17,18].

Generally, two carbonization methods can be employed, namely, hydrothermal carbonization or drying carbonization; the selection of these methods impacts consumption energy, recovery rate, and final product properties. Recently, flash pyrolysis with thermal shock has emerged as a promising carbonization technique, offering rapid heating and efficient pore development [19]. Third, the two different activation methods are physical activation and chemical activation. Physical activation uses steam or CO_2_ as activating agents, whereas chemical activation uses chemicals such as KOH, K_2_CO_3_, H_3_PO_4_, and ZnCl_2_ [20]. Physical activation is a simpler process but limited by a low recovery yield, high consumption energy, and the sensitivity of raw materials compared to chemical activation. Conversely, chemical activation leads to a higher surface area; therefore, biomass raw materials with low homogeneity are typically activated via chemical activation [21].

The importance of activation conditions has been highlighted in numerous studies. Such studies have investigated the effects of different activation agents on the porosity and adsorption properties of biomass-derived activated carbon. For instance, Pal et al. synthesized waste palm trunk-based activated carbon using KOH activation at 900 °C, achieving a specific surface area exceeding 2900 m^2^/g [22]. Minakshi et al. synthesized mango seed-based activated carbon using KOH activation at 800–1100 °C, achieving a specific surface area exceeding 1900 m^2^/g. Their findings highlight the crucial role of activation temperature and activator ratio in determining the final pore structure and adsorption performance of the carbon material and the need for process optimization to achieve high surface area and enhanced adsorption properties [23].

Importantly, the manufacturing method is ultimately reflective of the resultant product. The current cost of BAC manufacturing by physical activation is USD 1000–2000/ton, which results in a specific surface area of 1000–1500 m^2^/g, whereas that employing chemical activation is USD 1400–2500/ton, with an improved specific surface area of 2000–3000 m^2^/g [24]. Thus, the demand for high-surface-area adsorbents has made the BAC manufacturing process more complex, and limited research has been conducted on a laboratory scale.

Consequently, researchers are investigating alternative raw materials and activation techniques to enhance the efficiency of BAC production while minimizing costs and energy consumption. Among these, SCG has emerged as a promising precursor for activated carbon due to its abundance, economic viability, and environmental advantages.

SCG has been shown to be an excellent raw material for the production of adsorbents because it is a cost-effective and environmentally-friendly carbon-based precursor [25,26,27]. Specifically, activated carbon from SCG has been obtained using a chemical activation method, with KOH as the activating agent; the resultant material had a specific surface area of 1200 m^2^/g and adsorptive capacity similar to that of activated carbon when tested using similar methylene blue and chlorophenol [26]. In another study, activated carbon was prepared via the impregnation method using phosphoric acid; this study yielded a surface area of 721 m^2^/g and a high fraction of micropores [27]. Overall, the production of activated carbon with a high surface area from SCG remains a challenge. Nevertheless, activated carbon with a surface area of 2746 m^2^/g has been prepared using chemical activation by KOH and acid washing in HCl [8].

Despite these advances, systematic optimization of pretreatment conditions for SCG-derived activated carbon remains underexplored. Therefore, the aim of this study is to investigate the influence of various pretreatment conditions on the properties of activated carbon prepared from SCG. The following pretreatment conditions are examined: (1) drying holding time, (2) carbonization factors (method, temperature, and time), and (3) chemical activation factors (activator ratio, activation time, and activation temperature). Moreover, this study explores the viability of activated carbon prepared from SCG as an industrial adsorbent, considering its butane working capacity for n-butane.

## 2. Materials and Methods

### 2.1. Physical Properties

SCG samples were obtained from five different local coffee shops in Kyungido, Korea. First, the SCG was oven dried at 105 °C in a convection oven (LO-FP505, LKlab Korea Inc., Namyangju-si, Republic of Korea). Then, dried SCG was measured to detect the moisture content at different times. Proximate analysis was performed using American Standard Test Method (ASTM D1762-84) standards [28] to determine moisture, volatile matter, total ash, and fixed carbon contents using an oven (DF-3.5, Dae Heung Science Co., Incheon, Republic of Korea) [29]. Elemental analysis was performed by an EA analyzer (FlashSMART, ThermoFisher Scientific Inc., Waltham, MA, USA) to determine the fraction of carbon (C), hydrogen (H), oxygen (O), nitrogen (N), and sulfur (S). Sample weight loss was detected via thermogravimetric analysis (TGA, TGA 4000, PerkinElmer Inc., Waltham, MA, USA). The specific surface area (m^2^/g) and porosity (micropore (cm^3^/g), mesopore (cm^3^/g), and total pore volume (cm^3^/g)) of activated carbon were determined by N_2_ adsorption/desorption at −196 °C using a Brunauer–Emmett–Teller analyzer (Trixtar II, Micromeritics, Norcross, GA, USA) and the t-plot method. Energy consumption for the two carbonization methods was measured with a power meter (HPM-100A, Wattman, Seoul, Republic of Korea).

The morphologies of prepared activated carbons were observed using field emission scanning electron microscopy (Mira 3, Tescan, Brno, Czech). The crystal structure was analyzed via X-ray diffraction (SmartLab SE, Rigaku, Tokyo, Japan) and Raman spectra (RAMANtouch, Nanophoton, Seattle, WA, USA). XRD was measured using a Cu Kα (1.542 Å) source over a 20–80° range. The Raman spectra were measured in the range of 0–4000 cm^−1^ at room temperature and a wavelength of 523.05 nm.

### 2.2. Carbonization

Hydrothermal carbonization was performed in a 500 cm^3^/batch reactor with temperature control. For samples with drying pretreatment, 100 g of SCG was loaded into the reactor, and then 235 mL of water was added. The reactor was closed under N_2_ and heated using an electric heater to the required temperature (180–250 °C) based on previous research [30]. The sample was then stirred using a four-blade stirrer for 1 h. The hydrolysate samples from the reactor were collected and filtered using a Whatman nylon membrane filter in a drying convection oven. The samples were denoted as HTC-“temperature” as follows: HTC-180, HTC-200, HTC-220, and HTC-250. For drying carbonization, the dried SCG was loaded into a 1 kg/batch reactor with 1 L/min of N_2_ for 30 min to maintain the inner condition. Subsequently, the temperature was increased to 300–700 °C and maintained for 1, 3, or 5 h. The resulting sample names are shown in Table 1.

### 2.3. Activation

Preparation of BAC from SCG was performed via chemical activation. The dried SCG and activating agent (KOH) were physically mixed and set in a stainless-steel reactor under nitrogen gas at 1 L/min for 30 min. The sample was activated at temperatures of 750–950 °C for 1–3 h at a heating rate of 5 °C/min. Upon completion of the activation process, the nitrogen flow was replaced with carbon dioxide at 1 L/min, and the CO_2_ flow was maintained until the reactor cooled to room temperature. The sample was then washed several times with deionized water via sonication until the pH of the filtrate became neutral. The washed samples were dried using hot-air convection (LO-FP505, LKlab KOREA Inc., Republic of Korea) to prepare the activated carbon. Three parameters affecting the properties of activated carbon were varied, including the chemical activation temperature (T = 750 °C, 850 °C, 950 °C), activation time (H = 1 h, 2 h, 3 h), and activator weight ratio (activator/precursor ratio = 1, 2, 3). Therefore, the activated carbon samples derived from chemical activation with KOH were denoted as CAC1–CAC9, as shown in Table 2.

### 2.4. Adsorption Efficiency

To evaluate adsorption efficiency, butane was used as an adsorbate. Stock standard gas of n-butane containing 0.3 g of activated carbon with 99.9% purity (JC GAS Co., Gwangju, Republic of Korea) was prepared and stored in U-type glass at 25 °C. The flow rate of n-butane gas was 250 mL/min for 15 min, then 250 mL/min for 10 min, before purging the N_2_ gas (99%) at 300 mL/min for 40 min until no more weight change was observed. This procedure was performed according to American Standard Test Method (ASTM D5228) standards [31]. All measurements were conducted in triplicate, and the reported values represent the average of these three repeated cycles.

The butane activity (BA), butane retentivity (BR), and butane working capacity (BWC) were estimated from the following equations:

(1) $$Butane\;activity\left(BA\right)=\frac{The\;weight\;of\;fully\;adsorbed\;cell-The\;weight\;of\;purged\;cell}{The\;weight\;of\;sample}\times 100$$

(2) $$Butane\;retentivity\;(BR)=\frac{The\;weight\;of\;fully\;adsorbed\;cell-The\;weight\;of\;loaded\;cell}{The\;weight\;of\;sample}\times 100$$

(3) $$Butane\;working\;capacity\left(BWC\right)=\frac{The\;weight\;of\;purged\;cell-The\;weight\;of\;loaded\;cell}{The\;weight\;of\;sample}\times 100$$

## 3. Results and Discussion

### 3.1. SCG Drying Conditions

**Figure 1:**
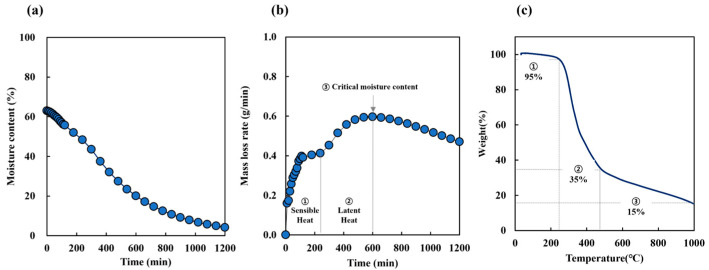
The physical properties of SCG: (**a**) moisture content, (**b**) mass loss rate, (**c**) thermogravimetric analysis (TGA).

The initial moisture content of SCG was measured at 50–60% (Table 3). Figure 1a presents the decrease in moisture content over drying time, while Figure 1b shows the corresponding mass loss rate, representing the rate at which mass decreased over time. During the early drying stage (0–150 min), the surface moisture evaporated rapidly, resulting in a high mass loss rate. Between 150 and 240 min, the process entered a transitional phase where the mass loss rate temporarily plateaued as heat gradually penetrated the interior of the sample. After 240 min, the internal moisture began evaporating more actively, leading to a renewed increase in the mass loss rate, which peaked around 600 min. Although the rate declined beyond this point, approximately 20% residual moisture remained, indicating that complete drying was not achieved within the observed timeframe. These results highlight the multi-stage drying dynamics of SCG and underscore how the mass loss rate reflects the combined effects of surface evaporation, internal heat transfer, and latent heat-driven moisture removal.

Accordingly, the theoretical energy consumption in convection was calculated as follows:

E_t_ (kWh) = Aνρ_a_tC_a_ΔT (4)

E_kg_ = E_t_/w_o_ (5)

where E_t_ is the total energy consumption (kWh), A is the cross-sectional area of the container, ν is the air velocity (m/s), ρ_a_ is the air density (kg/m^3^), t is the total drying time (h), C_a_ is the specific heat (kJ/kg°C), ΔT is the temperature difference (°C), E_kg_ is the specific energy required, and wo is the initial weight of the sample (kg). Therefore, owing to the high moisture content of the biomass, energy consumption during SCG drying was 49 kWh/kg, which is similar to the specific energy requirement for convection drying (51 kWh) [32].

Omitting the carbonization of biomass precursor results in a low product yield [14]; therefore, most processes require carbonization to increase the carbon fraction. To select the carbonization temperature, TGA was performed (Figure 1c). The resulting thermal profile indicated three states: dehydration, devolatilization, and solid decomposition. The first stage of weight loss up to 220 °C corresponded to the moisture content of the raw materials (Figure 1b). The second stage of weight loss, between 220 °C and 450 °C, may be attributed to the decomposition of hemicellulose and the release of volatile matter in the SCG. The third stage, observed beyond 450 °C, is attributed to the gradual decomposition of lignin, which exhibits high thermal stability, as well as the rearrangement and condensation of the carbon matrix. This stage leads to the formation of a thermally stable carbon structure and accounts for the residual char observed at the end of the thermal analysis. Therefore, the drying carbonization temperature was set between 300 °C and 700 °C. For hydrothermal carbonization, the temperature range was set between 160 °C and 250 °C, based on our previous research [30].

Finally, the SCG composition in wt.% carbon, hydrogen, oxygen, nitrogen, and sulfur is shown in Table 4. The ratio of hydrogen/carbon was approximately 0.13 for all raw SCG samples. Generally, a lower H/C ratio indicates a higher carbon content and greater thermal stability, which can result in a stable carbon layer. This also means less carbon loss during activation, making it suitable as a precursor for activated carbon. Therefore, from the various SCG samples shown in Table 1, SCG #3 was selected for subsequent experiments owing to its lower H/C ratio.

### 3.2. Carbonization Properties

#### 3.2.1. Hydrothermal Carbonization

**Figure 2:**
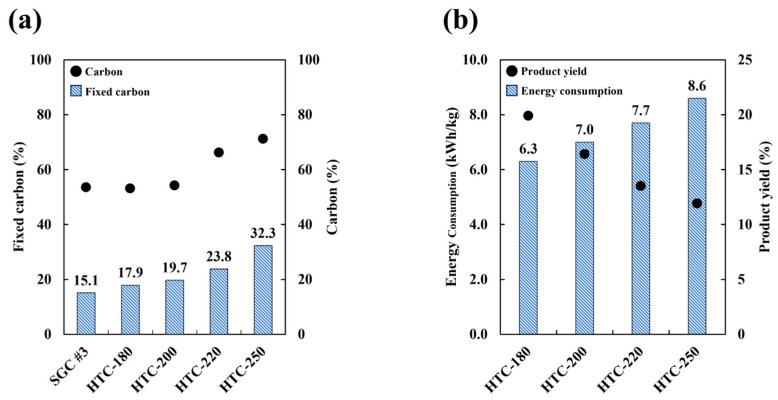
(**a**) The physical properties (fixed carbon and carbon fraction) of HTC samples and (**b**) the consumption energy and product yield after HTC.

As temperature is one of the primary determinants of product properties, its effect was investigated through hydrothermal carbonization experiments conducted at 180–250 °C for 1 h at a feedstock-to-water weight ratio of 1:2. The proportion of fixed carbon increased as the hydrothermal carbonization temperature increased (Figure 2a), to 15–32% more than that in the raw materials. According to elemental analysis, the carbon fraction also increased with increasing hydrothermal temperature, but the rate of increase was low. Finally, as observed in Figure 2b, energy consumption was in the range of 6.3–8.6 kWh/kg, and it increased with the hydrothermal temperature, followed by a decrease in product yield. Therefore, the increase in carbon fraction was associated with higher energy consumption and a reduction in product yield.

#### 3.2.2. Drying Carbonization

**Figure 3:**
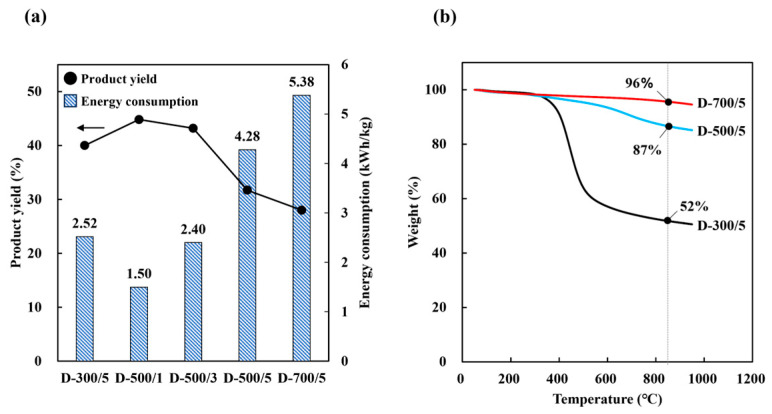
(**a**) Solid yield and consumption energy with different drying carbonization methods and (**b**) TGA after carbonization.

Thermal analysis was performed to determine the thermal behavior of the product under carbonization. Table 5 shows the proximate and elemental analysis of dried SCG subjected to drying carbonization under different temperature and time conditions. The amount of fixed carbon increased with increasing carbonization temperature and time. The H/C atomic ratio generally decreased with heating temperature and time, which was consistent with the reported increasing aromaticity (=H/C) [33]. This means that the stability of biochar was enhanced as an adsorbent material, which is consistent with our research findings, where stability improves as the reaction temperature increases [34]. The carbonization temperature had a greater effect on the rate at which fixed carbon increased than time. Table 5 and Figure 3a show that the fixed carbon fraction and energy consumption were proportional to the carbonization temperature and time, but the product yield was inversely proportional to these parameters. Based on the TGA results, different thermal behavior was observed with different carbonization temperatures (Figure 3b). Specifically, the amount of solid residue at the end of the process increased with increasing carbonization temperature. During the carbonization process of SCG, the first stage of weight loss associated with hemicellulose decomposition was not observed for D-500/5 and D-700/5. During carbonization, the thermal decomposition of hemicellulose and cellulose at 200~400 °C led to the release of volatile compounds (e.g., CO_2_, CO, CH_4_), creating initial pore structures within the carbon matrix. At higher temperatures (400~600 °C), the gradual degradation of lignin and the rearrangement of fixed carbon further stabilized the porous framework. These physicochemical transformations collectively contributed to the development of both micropores and mesopores, enhancing the overall specific surface area and adsorption performance of the final material.

Based on energy consumption, the proportion of fixed carbon, and the product yield values obtained, it is obvious that successful dry carbonization could be achieved by maintaining carbonization temperature at 500 °C for 1 h. Furthermore, drying carbonization is suitable for SCG carbonization because it can result in high product yields and increased amounts of fixed carbon with lower consumption energy than hydrothermal carbonization.

### 3.3. Properties and Characteristics of Chemically Activated Carbon

#### 3.3.1. Morphology of Chemically Activated Carbon

Figure S1 presents the SEM images of SCG, D-500/1, and CAC samples (3, 6/1 h, 6, and 9). The SCG sample exhibited a relatively dense and compact structure with smooth surfaces and minimal porosity. The higher-magnification image of D-500/1 confirms that the carbonization process effectively removed non-carbon components. A comparative analysis of D-500/1, CAC 3, 6/1 h, and 6 indicates that chemical activation significantly enhanced pore formation and surface roughness. The well-developed honeycomb-like porous network observed in CAC 6 further supports the notion that chemical activation markedly improves surface area and adsorptive properties. Conversely, CAC 9 exhibited significantly reduced particle size with inadequate pore formation, suggesting suboptimal activation conditions.

#### 3.3.2. Microstructural and Textural Properties

**Figure 4:**
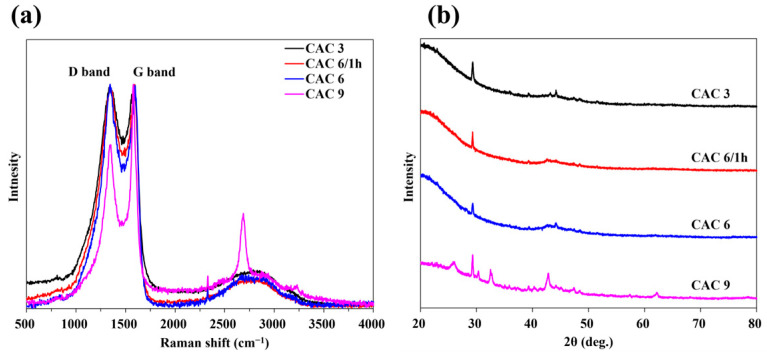
The (**a**) Raman spectra and (**b**) X-ray diffraction patterns of CAC 3, 6/1 h, 6, and 9.

Figure 4 presents the Raman spectra and XRD patterns of CAC 3, 6/1 h, 6, and 9. The Raman spectra of CAC 3, 6/1 h, and 6 exhibited characteristic features of activated carbon (AC), including the D-band (~1350 cm^−1^) and G-band (~1580 cm^−1^). The intensities of the D-band and G-band in these samples were relatively balanced, indicating an appropriate level of disorder while preserving the graphitic structure. In contrast, CAC 9 displayed not only the D-band and G-band but also the 2D-band (~2700 cm^−1^). The Raman spectra of CAC 9 closely resemble those of reduced graphene oxide (rGO) [35], suggesting an increase in carbon crystallinity as activation progressed at high temperatures.

The broad diffraction peak around 20–25° (2θ) in XRD pattern is characteristic of amorphous carbon, reflecting the disordered carbon structure present in CAC3, CAC6/1 h, and CAC6. In contrast, CAC9 exhibited a sharper peak near 25°, indicating the development of graphitic domains and partial graphitization due to high-temperature activation. Minor additional peaks are attributed to residual inorganic compounds such as K_2_CO_3_ formed during the KOH activation process.

Several studies have reported the transformation of porous graphene-based materials through high-temperature heat treatment utilizing chemical activation agents as catalysts [36,37]. In the present study, the CAC9 sample underwent heat treatment at 950 °C, a higher temperature than that of other samples, which resulted in enhanced crystallinity and the progression of graphene-like structural formation within the porous framework. This trend was further corroborated by the comparison of Raman and XRD spectra. Notably, the sharp peak observed around 25° in the XRD pattern of the CAC9 sample suggests a transition from amorphous carbon to a more crystalline structure.

**Figure 5:**
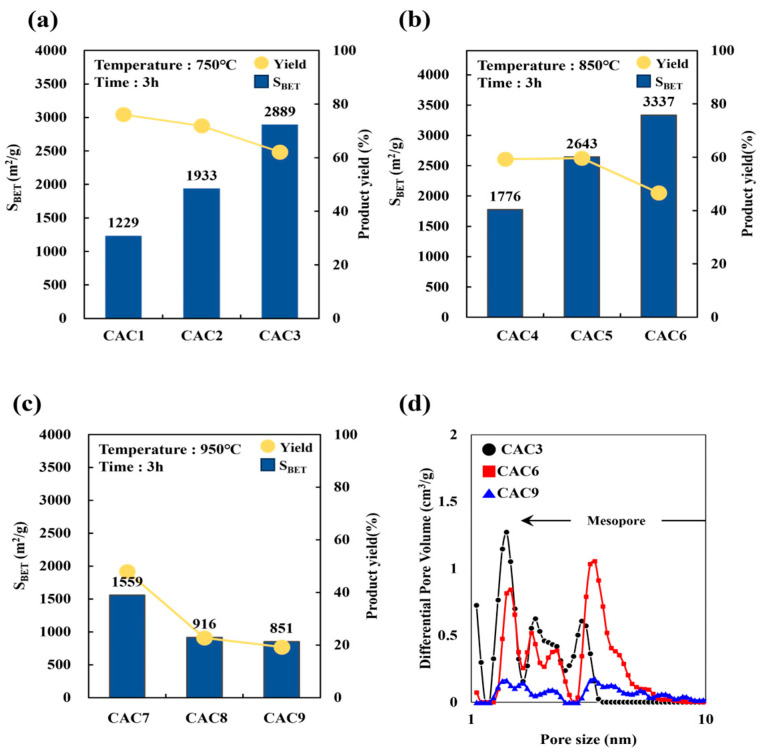
The surface area and product yield with different activation temperatures (**a**) 750 °C, (**b**) 850 °C, (**c**) 950 °C for 3 h, and the (**d**) pore size distribution of CAC3, CAC6, and CAC9.

Table 6 and Figure 5 display the specific surface area and volumes of chemically activated carbon (CAC) samples according to different activation temperatures, times, and agent ratios for D-500/1 samples. After chemical activation, a significant increase in surface area was observed with increasing temperature and activation agent ratio, to more than two times that before chemical activation. The maximum specific surface area was achieved with an activation agent ratio of 3 and an activation temperature and time of 850 °C and 3 h, respectively (CAC6). Unexpectedly, the specific surface areas of CAC7–CAC9 decreased with increasing activated agent ratio, an outcome that may be attributable to collapsed pores [38,39,40].

The micropore volume of CACs decreased with increasing activation agent ratio and activation temperature, whereas their mesopore volume increased until the activation temperature reached 850 °C, then decreased at higher temperatures. This indicates that temperatures higher than 850 °C led to pore collapses [20], likely due to excessive chemical activation under high KOH ratios and temperatures, which over-etches the carbon framework and causes pore wall collapse. This structural degradation reduces the effective micropore and mesopore network, ultimately lowering the available surface area for adsorption.

The BAC product yield of CAC1–CAC9 decreased from 76% to 19% with increasing temperature from 750 °C to 950 °C and activation agent ratio from 1 to 3, primarily due to increased carbon loss and volatilization under over-activation conditions. Thus, CAC6 was deemed the optimum condition for manufacturing low-cost, renewable BAC with a high surface area, balancing pore development and material integrity without sacrificing yield, which has implications for economic and environmental materials manufacturing. The surface area and pore volume measurements of CAC samples are presented in Figure S2. The isotherms of CAC3 and CAC6 corresponded to type I(b) (IUPAC classification), indicating the dense micropore–mesopore structure typical of activated carbon. However, the isotherms of CAC9 corresponded to type IV(a), which represents a dense mesopore–macropore structure [41]. This shift was likely due to the high-temperature-induced expansion and partial collapse of microporous frameworks, which enlarges pores into the mesopore and macropore ranges. Therefore, these results confirmed the production of mesoporous activated carbons with excellent adsorption characteristics [42].

**Figure 6:**
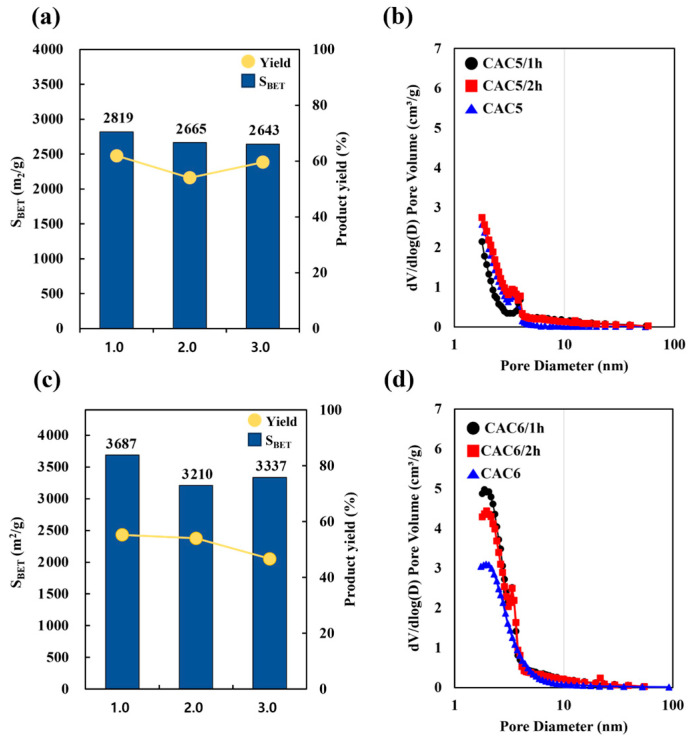
The (**a**) surface area, product yield, and (**b**) pore diameter of the CAC5 groups (agent ratio 2 at 850 °C for 1 h to 3 h), and the (**c**) surface area, product yield, and pore diameter of the CAC6 groups (agent ratio 3 at 850 °C for 1 h to 3 h), and the (**d**) pore diameter of the CAC6 groups (agent ratio 3 at 850 °C for 1 h to 3 h).

Table 7 and Figure 6 compare the physical properties of CACs for different activation times from 1 h to 3 h at 850 °C and an activation agent ratio of 2 (CAC5 groups) and 3 (CAC6 groups), which represent the optimum conditions. As the agent ratio varied between the CAC5 and CAC6 groups, the surface area did not depend on the activation time. For both CAC5 and CAC6 groups, no significant increase in the surface area (3210–3687 m^2^/g and 2643–2819 m^2^/g, respectively) or product yield (60–62% and 54–59%, respectively) was observed with increasing activation time. These results further confirmed the production of mesoporous activated carbon with excellent adsorption characteristics, with mesopore fractions of 40% and 72% for CAC5 and CAC6 groups, respectively.

Regarding the pore diameter and pore volume, the CAC5 groups showed similar results to the CAC6 group at an activation agent ratio of 3, even when the activation time was increased. In the CAC6 group, the micropore volume decreased rapidly as the activation time increased, and the peak in the region of 2–3 nm disappeared. The surface area and pore volume measurements are presented in Figure S3. The isotherms of the CAC5 and CAC6 groups corresponded to type I(b) (IUPAC classification), which indicates the dense micropore–mesopore structure typical of activated carbon. In this study, the high surface area (3687 m^2^/g) and high mesopore volume fraction (68%) of the prepared CAC6 samples indicate their applicability for gas-phase adsorption or gas storage.

As shown in Table 8, our SCG-derived activated carbon outperformed many biomass-based activated carbons in terms of surface area and mesoporous structure. Compared to previously reported SCG-based activated carbon (2746 m^2^/g) and mango seed-derived carbon (1944 m^2^/g), the optimized activation method in this study resulted in significantly higher porosity and mesopore volume (1.45 cm^3^/g), highlighting its enhanced adsorption potential. Additionally, the H_3_PO_4_-activated SCG (720 m^2^/g, 0.051 cm^3^/g mesopore volume) exhibited much lower porosity, further reinforcing the effectiveness of our KOH-based activation process. Furthermore, the H_3_PO_4_-activated coconut shell-derived carbon (891 m^2^/g, 0.46 cm^3^/g mesopore volume) and KOH-activated bamboo-derived carbon (3208 m^2^/g, 1.01 cm^3^/g mesopore volume) provide additional comparison points, demonstrating that while bamboo-derived carbon achieves a comparably high surface area, our SCG-based carbon exhibited superior mesoporous volume, making it highly suitable for applications requiring large pore structures. These results emphasize that the combination of optimized drying, carbonization, and chemical activation parameters enables the production of highly porous activated carbon suitable for industrial applications, particularly in gas-phase adsorption.

### 3.4. Butane Working Capacity Test

**Figure 7:**
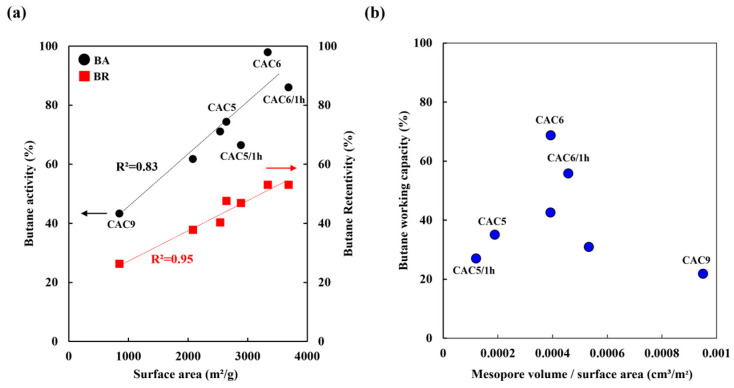
Butane working capacity of CACs as a function of activation conditions. (**a**) BA, BR of CACs as a function of surface area, (**b**) Correlation between mesopore volume-to-surface area ratio and butane working capacity.

The BWC test, the most common analytical method for analyzing the performance of activated carbon canisters, analyzes the adsorption and desorption behavior of activated carbon on n-butane. Figure 7 presents the calculated BA, BR, and BWC of CACs according to Equations (1)–(3), respectively. With increasing surface area, which provides more sites for physical adsorption and desorption, BA and BR increased from 43% to 98% and from 26% to 53%, respectively. The BA and BR of activated carbon have a close relationship with the sub-mesopore volume in the range of 1.5–3.8 nm and with the mesopore volume in the range of 5.0–7.0 nm, respectively [41,45]. However, the mesopore volume fraction was calculated based on the surface area, and the correlation with BWC was confirmed because the surface area affects adsorption and desorption. Thus, the BWC decreased when the fraction of mesopore volume/surface area was higher or lower than 0.0004 cm^3^/m^2^, and the mesopore volume/surface area of 0.0004 was the maximum. Under high-temperature chemical activation (950 °C), the mesopore volume fraction was high at a low surface area, resulting in low BWC. Accordingly, a mesopore volume-to-surface area ratio of approximately 0.0004, such as that in CAC6, exhibited the highest BWC performance.

According to Lee et al. [41], the butane working capacity (BWC) of the commercial activated carbons WVA-1100 (Ingevity, North Charleston, SC, USA) and 2GK (Kuraray, Osaka, Japan) was reported to be 35.0 wt.% and 37.7 wt.%, respectively. Additionally, Lee et al. reported that the BWC of BAX-1500 (Ingevity, USA) was 42.0 wt.% [42].

Table 9 presents a comparative analysis of the butane adsorption performance of CAC6, BAX-1500, WVA1100, and 2GK. The results indicate that CAC6 demonstrated a significantly higher BWC (74.6 wt.%) compared to commercial activated carbons. The superior BWC of CAC6 indicates that its optimized pore structure and activation conditions enhanced both butane adsorption and retention, suggesting its potential for high-performance adsorption applications. Additionally, the BWC values were averaged over three repeated cycles, confirming the sufficient reusability of CAC6.

## 4. Conclusions

In this study, various SCG processing methods (drying, carbonization, and chemical activation) were optimized to develop high-performance activated carbon with a specific surface area of 3687 m^2^/g and a butane working capacity (BWC) of 74.5%. Compared to previously reported biomass-derived carbons, such as coconut shell-based (891 m^2^/g) [43], mango seed-based (1944 m^2^/g) [23], and bamboo-based activated carbon (3208 m^2^/g) [44], the material developed in this study exhibited superior mesoporosity and competitive adsorption performance, underscoring the effectiveness of the optimized pretreatment and activation strategies. Notably, the overall yield of 10.6% from raw SCG, coupled with high mesoporosity, highlights the material’s practical potential for industrial applications. Optimal processing conditions were identified as follows:

Drying: Stable drying was carried out over 20+ h at 105 °C in a convection oven with an energy input exceeding 49 kWh/kg, reducing moisture content below 57.1% (yield: 42.9%).

Carbonization: Drying carbonization was carried out at 500 °C for 1 h under an inert N_2_ atmosphere, yielding 44.8% carbonized SCG.

Chemical Activation: KOH activation was performed at 850 °C for 1 h with a 3:1 agent ratio, achieving a final surface area of 3687 m^2^/g, a mesopore volume of ≥70%, and an overall yield of 10.6% from raw SCG.

These results confirm the suitability of the synthesized activated carbon for canister and gas-phase adsorption applications. This work provides a scalable and energy-efficient pathway for transforming low-cost, renewable SCG waste into high-performance adsorbent materials, promoting sustainable waste valorization and circular economy practices.

## Figures and Tables

**Table 1:** The denoted names of the carbonization samples.

Name	H-180	H-200	H-220	H-250	D-300/5	D-500/1	D-500/3	D-500/5	D-700/5
Carbonization method	Hydrothermal				Drying				
Temperature (°C)	180	200	220	250	300	500	500	500	700
Time (h)	1	1	1	1	5	1	3	5	5

**Table 2:** The denoted name of the activated carbon depends on the different chemical activation conditions.

Name	CAC 1	CAC 2	CAC 3	CAC 4	CAC5/1 h	CAC5/2 h	CAC 5	CAC6/1 h	CAC6/2 h	CAC 6	CAC 7	CAC 8	CAC 9
Temperature (°C)	750	750	750	850	850	850	850	850	850	850	950	950	950
Time (h)	3	3	3	3	1	2	3	1	2	3	3	3	3
Agent ratio (*w*/*w*)	1	2	3	1	2	2	2	3	3	3	1	2	3

**Table 3:** Moisture content of spend coffee grounds.

Sample Number	#1	#2	#3	#4	#5	Average
Moisture contents (%)	57.0	56.0	64.6	43.0	65.0	57.1 ± 8.9

**Table 4:** The proximate analysis and elemental analysis of dried SCG.

Sample Number		#1	#2	#3	#4	#5	Average
Proximate Analysis (wt%)	Volatiles	88.4	82.6	87.5	80.4	84.5	84.7 ± 3.3
	Fixed-C	11.0	16.4	17.6	17.5	13.4	15.2 ± 2.9
	Ash	0.6	1.0	0.9	1.9	2.1	1.3 ± 0.7
Elemental analysis (wt%)	C	50.2	51.8	54.1	55.2	53.5	53.0 ± 2.0
	H	7.2	7.2	6.7	7.1	7.1	7.1 ± 0.2
	O	39.1	37.7	32.7	32.9	33.9	35.3 ± 2.9
	N	2.3	2.2	2.6	2.4	2.3	2.4 ± 0.2
	S	0	0	0	0	0	0
H/C (-)		0.14	0.14	0.12	0.13	0.13	0.13

**Table 5:** The proximate analysis and elemental analysis depend on different drying carbonization methods for dried SCG.

Samples		D-300/5	D-500/1	D-500/3	D-500/5	D-700/5
Proximate analysis (wt%)	Volatiles (VR)	59.2 (▼32%)	12.9 (▼85%)	12.8 (▼85%)	11.7 (▼87%)	7.4 (▼92%)
	Fixed-C (CR)	21.9 (▲24%)	84.8 (▲382%)	84.2 (▲378%)	83.7 (▲376%)	84.5 (▲380%)
Elemental analysis (wt%)	C	71.9	83.3	82.6	85.2	88.9
	H	6.9	2.8	2.8	2.7	1.2
H/C (-)		0.09	0.03	0.03	0.03	0.01
Yield (%)		40.0	44.8	43.2	31.7	28.0

VR: volatile rate (%) = (volatile content of carbide − volatile content of precursor)/volatile content of precursor × 100, CR: fixed-C rate (%) = (fixed carbon content of carbide − fixed carbon content of precursor)/fixed carbon content of precursor × 100, ▼: decrease compared to SCG, ▲: increase compared to SCG.

**Table 6:** The physical properties of CACs with different activation temperatures (750–950 °C) and agent ratio (1 to 3) after drying carbonization at 500 °C for 1 h.

Sample	CAC 1	CAC 2	CAC 3	CAC 4	CAC 5	CAC 6	CAC 7	CAC 8	CAC 9
S_BET_ (m^2^/g)	1230	1933	2889	1776	2643	3337	1559	916	851
V_T_ (cm^3^/g)	N.A.	N.A.	1.29	N.A.	1.28	1.95	N.A.	N.A.	0.84
V_MES_ (cm^3^/g)	N.A.	N.A.	0.35	N.A.	0.50	1.53	N.A.	N.A.	0.80
V_MIC_ (cm^3^/g)	N.A.	N.A.	0.94	N.A.	0.78	0.42	N.A.	N.A.	0.04
Yield (wt%)	76.1	71.9	62.1	59.3	59.6	46.7	47.9	22.7	19.2

N.A.: Not Analyzed—Analysis was not performed for this condition.

**Table 7:** The physical properties of CACs with different activation times (1 h to 3 h) and agent ratios (2 to 3) at 850 °C after drying carbonization at 500 °C for 1 h.

Sample	CAC 5/1 h	CAC 5/2 h	CAC 5	CAC 6/1 h	CAC 6/2 h	CAC 6
S_BET_ (m^2^/g)	2819	2665	2643	3687	3210	3337
V_T_ (cm^3^/g)	1.40	1.41	1.28	2.11	1.87	1.95
V_MES_ (cm^3^/g)	0.49	0.69	0.50	1.45	1.33	1.53
V_MIC_ (cm^3^/g)	0.91	0.73	0.78	0.66	0.54	0.42
Yield (wt%)	61.9	60.0	59.6	55.2	54.0	46.7

**Table 8:** Comparative textural properties of biomass-derived activated carbons.

Raw Material	Activator	S_BET_ (m^2^/g)	V_T_ (cm^3^/g)	V_MES_ (cm^3^/g)	Ref.
Spent coffee grounds	KOH	3687	2.11	1.45	This study
Waste palm trunk	KOH	2927	2.51	0.10	[27]
Mango seed	KOH	1944	0.39	-	[23]
Coconut shell	H_3_PO_4_	891	0.72	0.46	[43]
Bamboo	KOH	3208	1.01	-	[44]
Spent coffee grounds	H_3_PO_4_	720	0.46	0.051	[23]
Spent coffee grounds	KOH	2746	2.32	1.34	[8]

**Table 9:** Comparison of butane working capacity among CAC6, WVA1100, and 2GK.

Sample	Butane Activity (*w*/*w*%)	Butane Retentivity (*w*/*w*%)	Butane Working Capacity (*w*/*w*%)	Ref.
CAC 6	78.9	4.3	74.6	This study
BAX-1500	50.0	8.0	42.0	[42]
WVA-1100	35.2	0.2	35.0	[41]
2GK	43.5	5.8	37.7	[41]

## Data Availability

The original contributions presented in this study are included in the article. Further inquiries can be directed to the corresponding author.

## Supplementary Materials

The following supporting information can be downloaded at: https://www.mdpi.com/article/10.3390/molecules30122557/s1. Figure S1: The SEM images of (a) SCG, (b) D-500/1, (c) CAC 3, (d) CAC 6/1 h, (e) CAC 6, and (f) CAC 9. Figure S2: The isotherms with different activation temperatures of (a) CAC3-750 °C, (b) CAC6-850 °C, and (c) CAC9-950 °C for 3 h; Figure S3: The isotherms with different activation times: (a) CAC5 group and (b) CAC6 group.
