# Supplementary material for: Optimization of Activated Carbon Synthesis from Spent Coffee Grounds for Enhanced Adsorption Performance
Source: Molecules. 2025 Jun 12;30(12):2557. doi: 10.3390/molecules30122557 (PMC12196381; doi:10.3390/molecules30122557)
Supplement: Supplementary file 1 [file molecules-30-02557-s001.zip › molecules-3632186-supplementary.pdf]

Supplementary Materials

Optimization of Activated Carbon Synthesis from Spent Coffee Grounds for Enhanced Adsorption Performance

Geon-Woong Hyeon [1], Gi Bbum Lee [1], Da Jung Kang [1], Sang Eun Lee [1], Kwang Mo Seong [2] and Jung-Eun Park [1,*]

1 Bio Resource Center, Institute of Advanced Engineering, 175-28 Goan-ro 51 beon-gil, Baegam-myeon, Cheoin-gu, Yungin-si 17180, Gyeonggi-do, Republic of Korea; 7669woong@iae.re.kr (G.-W.H.); mnbbv21c@iae.re.kr (G.B.L.); kangdj1119@iae.re.kr (D.J.K.); lse9907@iae.re.kr (S.E.L.)

2 Sustainable Materials Research Team, Hyundai Motor Group, 37, Cheoldobangmulgwan-ro,

Uiwang-si 16082, Gyeonggi-do, Republic of Korea; aektu2000@hyundai.com

* Correspondence: jepark@iae.re.kr; Tel.: +82-31-330-7222

(a)

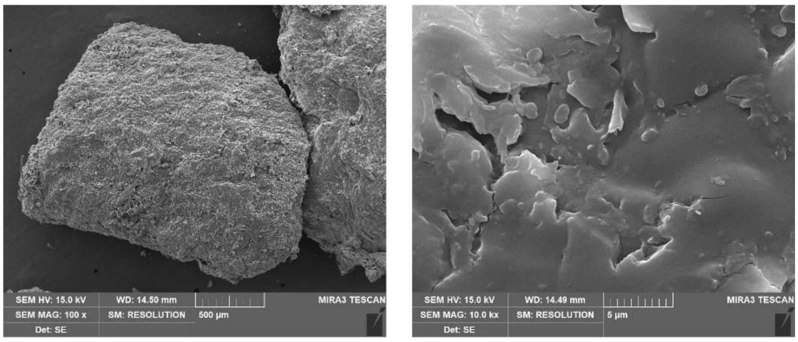
SEM HV: 15.0 kV
WD: 14.50 mm
MIRA3 TESCAN
SEM MAG: 100 x
SM: RESOLUTION
500 µm
Det: SE
SEM HV: 15.0 kV
WD: 14.49 mm
MIRA3 TESCAN
SEM MAG: 10.0 kx
SM: RESOLUTION
5 µm
Det: SE

(b)

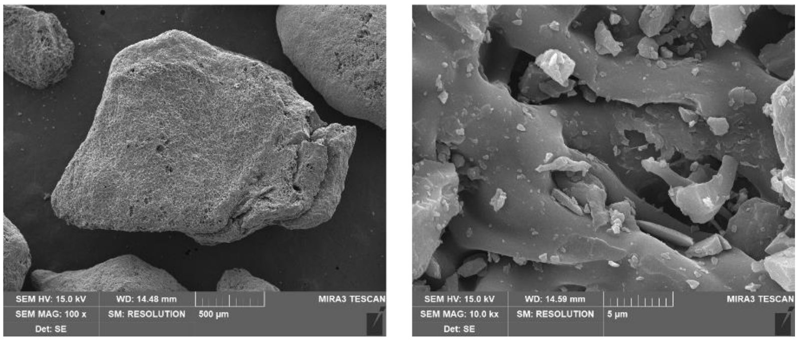
SEM HV: 15.0 kV
WD: 14.48 mm
MIRA3 TESCAN
SEM MAG: 100 x
SM: RESOLUTION
500 µm
Det: SE
SEM HV: 15.0 kV
WD: 14.59 mm
MIRA3 TESCAN
SEM MAG: 10.0 kx
SM: RESOLUTION
5 µm
Det: SE

(c)

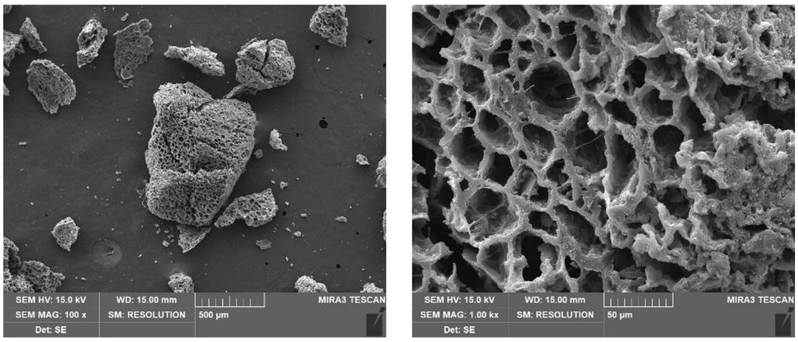
SEM HV: 15.0 kV
WD: 15.00 mm
MIRA3 TESCAN
SEM MAG: 100 x
SM: RESOLUTION
500 µm
Det: SE
SEM HV: 15.0 kV
WD: 15.00 mm
MIRA3 TESCAN
SEM MAG: 1.00 kx
SM: RESOLUTION
50 µm
Det: SE

(d)

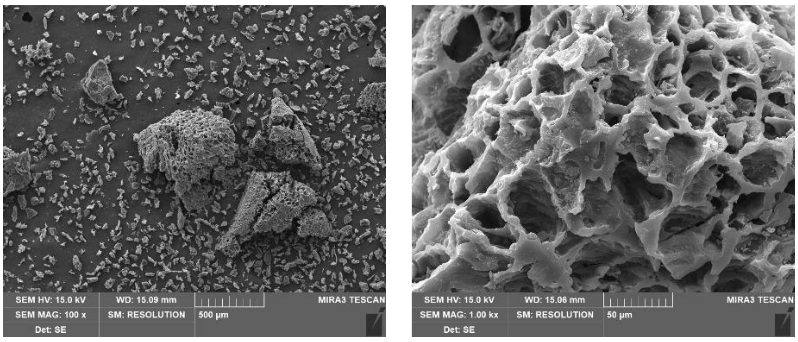
SEM HV: 15.0 kV
WD: 15.09 mm
MIRA3 TESCAN
SEM MAG: 100 x
SM: RESOLUTION
500 µm
Det: SE
SEM HV: 15.0 kV
WD: 15.06 mm
MIRA3 TESCAN
SEM MAG: 1.00 kx
SM: RESOLUTION
50 µm
Det: SE

(e)

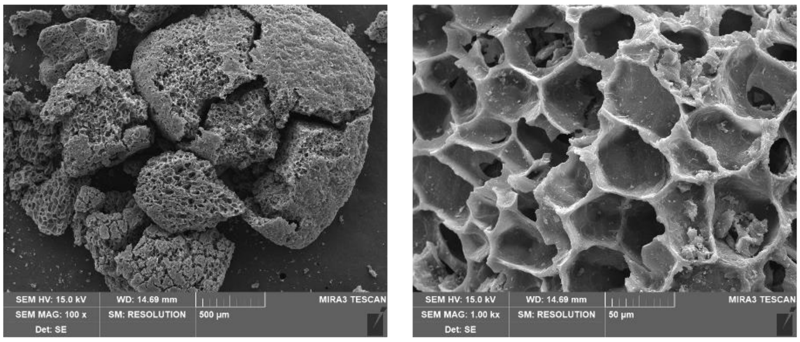

(f)

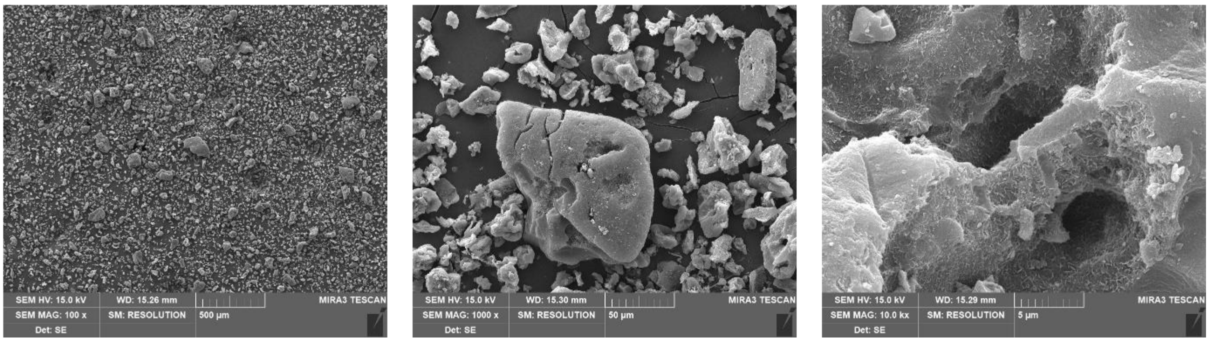

Figure S1. The SEM images of (a) SCG (b) D-500/1 (c) CAC 3 (d) CAC 6/1h (e) CAC 6 (f) CAC9

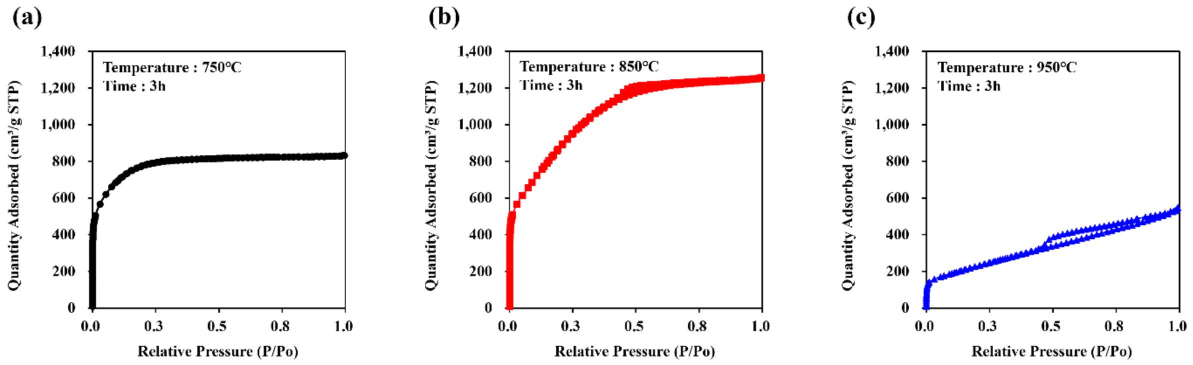

Figure S2. The Isotherms with different activation temperatures (a) CAC3-750°C, (b) CAC6-850°C, (c) CAC9-950°C for 3h

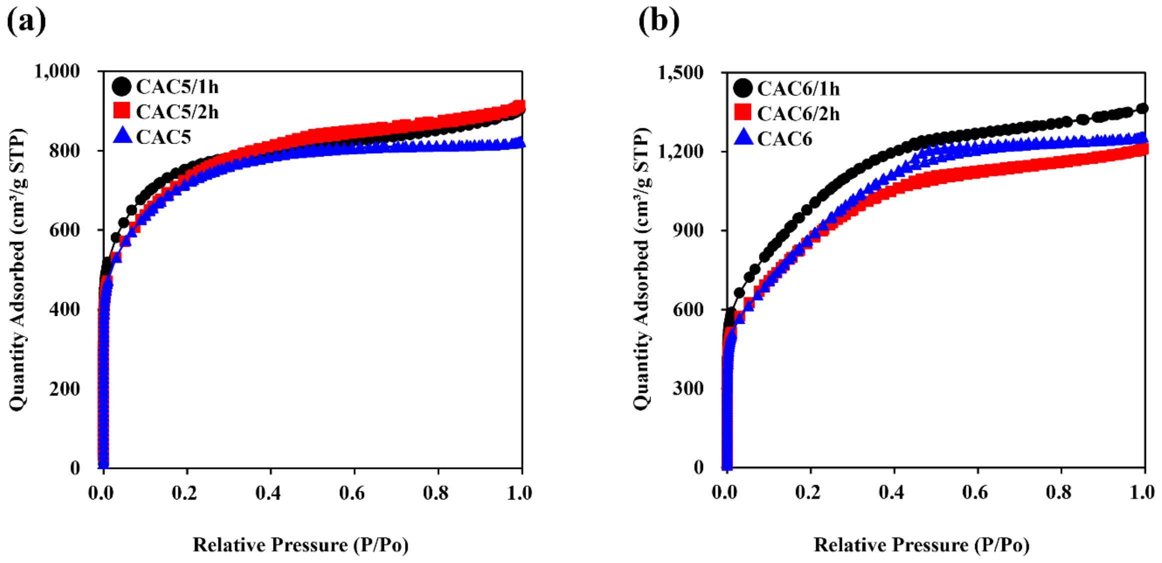

Figure S3. The Isotherms with different activation time (a) CAC5 group, (b) CAC6 group
